# Vitrification Increased Vacuolization of Human Spematozoa: Application of MSOME Technology

**Published:** 2017

**Authors:** Sara Taherzadeh, Mohammad Ali Khalili, Azam Agha-Rahimi, Fateme Anbari, Shahin Ghazali, Guido Macchiarelli

**Affiliations:** 1- Research and Clinical Center for Infertility, Shahid Sadoughi University of Medical Sciences, Yazd, Iran; 2- Department of Life, Health and Environmental Sciences, University of L’Aquila, L’Aquila, Italy

**Keywords:** MSOME, Sperm, Vitrification, Zona binding assay

## Abstract

**Background:**

Sperm vitrification is a technique of ice and cryoprotectant free cryopreservation by direct plunging of sperm suspension into liquid nitrogen (LN2). The aim of this study was to investigate the influence of cryoprotectant free-vitrification on human sperm fine structure by MSOME technology and the fertility potential by zona binding assay (ZBA).

**Methods:**

20 normo-ejaculates were prepared by swim up technique, and supernatants were divided into two parts of fresh and vitrified groups. For vitrification, sperm was dropped into LN2. Sperm motility, morphology, viability and MSOME were evaluated for each sample. In MSOM morphologically normal sperm (class 1), ≤2 small vacuoles (class 2), and one large vacuole or >2 small vacuoles (class 3) were evaluated. Also, fertility potential was evaluated by zona binding assay. Data was analyzed using paired t-test or Willcoxon’s test and p-value <0.05 was considered significant.

**Results:**

Vitrification significantly reduced both progressive motility, viability and morphology. Also, normal morphology of spermatozoa decreased significantly after vitrification. In MSOME evaluation, normal motile spermatozoa (Class 1) decreased from 23.00±12.44 to 16.00.56±10.79 after vitrification (p=0.008). Although spermatozoa classes 2 and 3 were increased, the difference was not significant. Moreover, fertility potential of motile spermatozoa was reduced after vitrification (9.0±13.87 *vs*. 13.40±22.73; p=0.07).

**Conclusion:**

Vitrification increased the rate of vacuolization in motile sperm head. Therefore, MSOME technology is recommended for assessment of sperm fine morphology in ICSI program used cryopreserved spermatozoa.

## Introduction

Sperm cryopreservation has been used to maintain fertility in cases with cancer or for reproductive medicine procedure. However, this technique may cause damages to the intracellular organelles and cell membrane with decrease in motility (1), morphology, viability and mitochondrial activity, by inducing various processes associated with cell death (2–4). Isachenko et al. noticed that vitrification free of cryoprotectant(CPA) can effectively preserve the cells with outcomes similar to conventional freezing method. In contrast to the programmable slow freezing, in vitrification, the intracellular ice crystallization was not formed removed and a glass-like (vitreous) state was formed at ultra-high speed cooling of the cells. It is known that spermatozoa contain large volume of proteins, sugars, and other components that may play as natural CPA(5). In our recent study, vitrification of normal spermatozoa showed similar results to rapid vapor freezing, with two advantages of absence of toxic effect of permeable CPA along with less DNA damage (6). It is still not clear whether vitrification can induce sperm nuclear damage or not. Sperm morphology determines the internal organelles such as vacuole, acrosome and base of head which have an important role in fertility. Most of the methods currently used to evaluate the fine structure of spermatozoa are invasive and may require fixation and fluorescent probes that endanger the sperm viability. However, the technique of motile sperm organelle morphology examination (MSOME) is non-invasive with real time assessment of motile spermatozoa. This high technology is successfully applied in intracytoplasmic selected sperm injection (IMSI) procedure (7). It is reported that IMSI procedure enhances the pregnancy rates and lowers the miscarriage rates when compared with ICSI (8, 9). However, some reports believed this technique does not improve pregnancy rate in male factor patients (10). It is reported that sperm vacuoles and chromatin immaturity have been associated with fertility potential of spermatozoa (11) and therefore MSOME is also useful to clarify the influence of vitrification on motile spermatozoa after warming. Furthermore, there is an important link between fragmented DNA or chromatin decondensation in spermatozoa with large vacuoles. Selection of motile, morphologically normal spermatozoa with no vacuoles or with 2 small vacuoles that account for less than 4% of the head’s cross sectional area for injection into the oocytes with IMSI resulted in higher rates of implantation and pregnancy, along with lower miscarriage rates when compared with conventional ICSI (12–15).

However, only few studies attempted to characterize the sperm abnormalities associated with sperm head vacuoles after cryopreservation. Recently, Boitrel et al. (2012) used the MSOME technology to assess the organelles of motile spermatozoa after slow freezing. They concluded that slow freezing alters the organelle morphology of motile human spermatozoa and induces sperm chromatin decondensation (16). There are, however, limited studies about the effect of cryopreservation, especially vitrification technique on sperm organelles. Therefore, the goal of this study was to investigate the influence of vitrification on fine structure of human spermatozoa using MSOME and fertility potential with zona binding assay (ZBA).

## Methods

The patients agreed to participate in this research via filling out the consent forms. The ethics committee of our institution approved this study.

### Samples:

The experiment was carried out on 20 normal ejaculates according to WHO 2010 (17). Semen samples of cases referred to our institute were prepared using direct swim up technique. Motile sperm fraction was diluted with Ham’s F10 medium supplemented with 5% human serum albumin (HSA) to reach final concentration of 20×10^6^/ *ml*. Each suspension was divided into two parts: I. Control (fresh) and II. Vitrification.

### Vitrification:

The suspension was mixed with 0.5 mol sucrose solution for 1:1 ratio at room temperature. Vitrification was done according to Isachenko’s protocol (18) using sperm final concentration of 10×10^6^. First, a vial was fixed in depth of the metal strainer (Cryo tube, 1.0 *ml*, NUNC) which was set in liquid nitrogen (LN). Then, 30 *μl* drop of sperm suspension was dropped into LN by micropipette. After solidification, the spheres were collected in a vial and kept in LN for 24 *hr*. For thawing procedure, each vial with solid spheres (up to 7) was solved into 5 *ml* Ham’s F10 with 5% HAS pre warmed to 37°*C* (warming media tube was immersed in 37°*C* water to stabilize the temperature), accompanied by vortexing for 5–10 *s*. Finally, the washing was done with centrifuging at 400 *g* for 5 *min* to concentrate the sperm cells. 0.5 *ml* Hams’F10 medium supplemented with 5% HSA was used for diluting the pellet. The post-thaw sperm suspension was maintained at 37°*C*/5% Co_2_ for 1 *hr*, and sperm analysis was done subsequently.

### Assessment of sperm parameters:

Sperm analysis was performed according to the WHO guideline (WHO, 2010). Briefly, for motility analysis, a wet preparation with a volume of 10 *μl* of sperm suspension was prepared. Two hundred spermatozoa per replicate were assessed for the percentage of different motile categories including progressive, non-progressive, and immotile.

Sperm vitality was determined using one-step eosin-nigrosin staining technique. Unstained spermatozoa were classified as live, while those showing pink or red color in the head region were considered dead. 200 spermatozoa were assessed for each preparation (17). For sperm morphology, 10 *μl* drop of semen was placed on a slide and a second slide was used to smear the sample in a thin distributed layer. The slide was air-dried, then fixed in ethanol-ether and papanicolaou staining. At least 200 spermatozoa were assessed for normal sperm morphology.

### Zona biding assay (ZBA):

Unfertilized human oocytes from ICSI cycles were incubated with 2×10^6^ motile sperm in 25 *μl* droplets of Ham’s F10 containing 5% HSA for 2 *hr* at 37°*C* in 5% CO_2_ in air. After 24 *hr* of incubation, the oocytes were transferred to Ham ’s F10 supplemented with 5% HSA and washed by repeated aspiration with a glass pipette to dislodge sperm loosely adhering to the surface of the ZP. The number of sperm bound tightly to the ZP was counted under×400 magnification using inverted microscopy.

Defective sperm–ZP binding is a clear indication of a severe sperm abnormality (19).

### MSOME:

**Figure 1.**
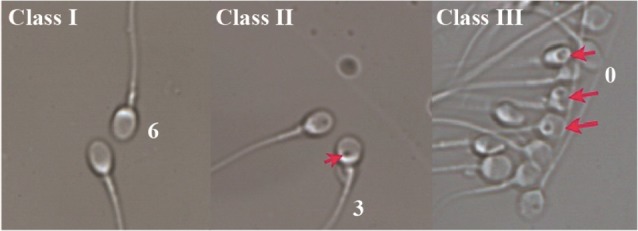
Spermatozoa at high magnification ×6000 with the aid of MSOME. A. sperm with class 1 without vacuole. B. sperm with class 2 with small vacuole. C. sperm with class 3 with large or multiple vacuoles

MSOME criteria for the morphologic normalcy of the sperm nucleus were defined according to Cassuto et al. (20). For observation up to ×6000, an aliquot (3 *μl*) of swim up sperm suspension was transferred to a 5 *μl* of 8% polyvinyl pyrrolidone (PVP; Irvine Scientific) under paraffin oil in a glass bottomed dish (WilCo-dish; WillCo Wells, Netherlands) and examined by inverted microscope (Eclipse TE 2000 Nikon, Japan) equipped with high-power differential interference contrast optics (DIC/Nomarski). Morphological evaluation was performed on the monitor screen, and 200 cells for each sample were assessed accordingly. The score of each spermatozoon was determined as: (2×Head)+(3×Vacuole)+(Base); (Normal head score=2), (Lack of vacuole score=3), (Normal base score=1) and (Total score =6) for a morphologic “normal top” spermatozoon. The scoring for each sperm ranged between 0 up to 6. Class 1 with high quality spermatozoa scored 4 to 6, class 2 was medium quality spermatozoa scored 1 to 3, and class 3 was low quality sperm scored 0 (20) (Figure 1).

### Statistical analysis:

Data are indicated as Mean± SD. Statistical analysis was performed with SPSS version 20. Student’s paired t-test or Willcoxon’s test were used for comparison of all parameters before and after vitrification depending on the normality of distribution of the variables.

## Results

### Sperm parameters:

There was a significant difference in the mean percentage of progressive, non-progressive immotility, and viability between the two groups. Light microscopic normal morphology using papanicolaou staining decreased significantly after vitrification (Table 1).

**Table 1.** Effects of vitrification on human sperm parameters

**Variables**	**Before**	**After**	**p-value**
**Progresive motility (%)**	88.39±12.29	39.80±10.62	<0.001
**Non-progressive motility (%)**	3.03±5.08	7.67±4.16	<0.001
**Total motility (%)**	91.43±10.64	47.46±10.99	<0.001
**Viability (%)**	85.90±13.18	49.20±18.31	<0.001
**Normal morphology (papanicolaou staining) (%)**	49.00±34.32	26.34±30.23	0.01

### Fertility potential:

The proportion of spermatozoa with fertility potential with ZBA test was lower after vitrification, when compared with control (9.0±13.87% *vs*. 13.40±22.73%, p=0.07) though this difference was not significant.

Motile sperm organelle morphology examination High score motile sperm (class 1) decreased significantly after vitrification (23.00±12.44% *vs*. 16.00±10.79%; p=0.008). However, there was not any significant differences between class 2 (55.26 ±11.45% *vs*. 59.31±11.73%) and class 3 (21.47± 15.54% *vs*. 24.68±10.97%) sperm morphology using MSOME after vitrification (Table 2).

**Table 2.** MSOME classification assessment of spermatozoa in fresh and vitrified samples

**MSOME**	**Fresh (%)**	**Vitrified (%)**	**p-value**
**Class 1**	23.00±12.44	16.00±10.79	0.008
**Class 2**	55.26±11.45	59.31±11.73	0.28
**Class 3**	21.47±15.54	24.68±10.97	0.31

## Discussion

Permeable CPA free vitrification is direct plunging of a sperm suspension into LN2. This technique avoids the use of the classic toxic permeable CPA that may have lethal effects of osmotic shock (5). The vitrification is an efficient and reliable technique for human sperm cryopreservation without requiring the CPAs (6). Previously, it was concluded that vitrification of neat ejaculates, particularly among abnormal semen, showed severe effect on sperm parameters (21). Therefore, sperm preparation could select best spermatozoa, preventing ROS production by dead or damaged spermatozoa.

There are several articles about the effects of vitrification on sperm parameters and DNA integrity. However, there is no study about the effect of this technique on sperm head vacuoles assessed by MSOME technology. It is not easy to monitor microvacuoles in the nucleus before ICSI by standard procedures and the intact paternal genome is required for proper embryo development. It is also not possible to assess real time DNA integrity and chromatin condensation before ICSI using staining procedure. It is, therefore, emphasized to apply a noninvasive method for detecting nuclear vacuoles in spermatozoa during ICSI. Cassuto et al. demonstrated a correlation between chromatin decondensation and score 0 sperm by high magnification morphology (22).

Origin of nuclear vacuoles remains a controversial issue. Some authors have shown that vacuole is the consequence of chromatin condensation in spermiogenesis. The quality of sperm chromatin-packaging seems to have a major impact on sperm morphology and early embryo development (15, 20). Recently, some reports concluded that application of MSOM and IMSI for sperm selection does not improve pregnancy rate in couples with male infertility (10, 23). Our results showed a significant decrease in the proportion of class1 after obtaining vitrified-warmed spermatozoa. Therefore, this study showed *in vitro* stresses, such as cryopreservation and induced sperm vacuolization. Although CPA free vitrification deleted the cryoprotectant cytotoxicity, it could decrease the high score sperm morphology. Lately, Gatimel et al. reported that slow freezing dose not elevate vacuolization in spermatozoa (24). On the other hand, Biotrelle et al. declared a decreasing in the number of motile spermatozoa and inducing vacuolization after slow freezing (16). In our recent study, it was reported that the ultra-structure of sperm head was changed after vitrification. Chromatin in fresh samples was more condense and there were few holes named “nuclear vacuole”. The number of nuclear vacuoles was increased and chromatin may have granular view (25). Also, in this study, abnormal morphology using papanicolaou staining increased after vitrification especially in head-tail category. This means that both head and tail morphology become more abnormal after vitrifiaction. It is believed that sperm vitrification caused damage to both tail and head regions of spermatozoa in both light microscopy and MSOME technique. In MSOME, only motile spermatozoa were surveyed, and this confirms that even spermatozoa with normal motility can be damaged following vitrification.

Another factor surveyed in this study was fertility potential of spermatozoa with ZBA assay. This assay highlights the potential of proper sperm interaction with oocyte *in vitro* setting. ZBA assay showed an insignificant decrease rate of sperm fertility endurance after vitrification. Evidence suggests that some spermatozoa intracellular signaling pathway can be affected during cryopreservation, and after warming of spermatozoa, features commonly observed in capacitated spermatozoa can be displayed (26, 27). It is, however, necessary to precisely detect the sperm fertility poteintial with application of fluorescent staining, in conjunction with ZBA. It was recently observed that the acrosome of many sperm are damaged after vitrification (25). However, in this assay, the concentration of motile spermatozoa was similar in both fresh and vitrified spermatozoa. Therefore, the fertilization potential of sperm could preserve their progressive motility after vitrification and it was similar to fresh spermatozoa. Defects in plasma membrane including rupture, loss, wrinkling, in addition to acrosomal abnormalities, such as distended structures, loss, and vesicle formations were reported previously (28).

## Conclusion

Vitrification had adverse effects on sperm parameters of motility and papanicolaou stained morphology. Also, it increased the rate of vacuolization in sperm head detected by MSOME, which may interfere with fertilization process in clinical setting. Therefore, MSOME technology is recommended for assessment of sperm fine morphology in ICSI program used cryopreserved spermatozoa.
